# Retraction: *Lycium barbarum* polysaccharide induced apoptosis and inhibited proliferation in infantile hemangioma endothelial cells via down-regulation of PI3K/AKT signaling pathway

**DOI:** 10.1042/BSR-2019-1182_RET

**Published:** 2024-05-08

**Keywords:** apoptosis, HemECs, Infantile hemangioma, Lycium barbarum polysaccharide, PI3K/AKT signal pathway

This article is being retracted from *Bioscience Reports* at the request of the Editor-in-Chief and the Editorial Board following receipt of a notification from a reader, alerting the Editorial Board to concerns surrounding the appearance of Western blots and flow cytometry plots in this paper. The Western blots have concerning features including the shapes of the bands and minimal background detail, and the flow cytometry scatter plots share similarities with those found in other papers by different authors. Additionally, images in this paper seem to appear in papers by different authors, specifically:

- Figure 2A 0 µg/mL panel which appears in Figure 6D by Chen et al, 2020 (DOI: 10.2147/CMAR.S243767) with rotation.
- Figure 5A IGF-1 panel which appears as Figure 7A Kasumi-1 Inhibitor panel by Piao et al, 2020 (DOI: 10.3892/ijmm.2020.4671) with rotation.

The authors have been contacted with regards to the retraction and have not responded to the Journal's queries or the concerns raised. Given the extent of the issues raised, the Editorial Board stand by the decision to retract the article.

